# Assessing Capacity and Implementation Status of the Disaster Risk Management Strategy for Health and Community Disaster Resilience in Malawi

**DOI:** 10.1007/s13753-021-00369-z

**Published:** 2021-10-08

**Authors:** Ozius Dewa, Donald Makoka, Olalekan A. Ayo-Yusuf

**Affiliations:** 1 Southern Africa Resilience Innovation Lab (SARiLab), School of Health Systems and Public Health, University of Pretoria, Pretoria, 0002 Gauteng South Africa; 2 Centre for Agricultural Research and Development, Lilongwe University of Agriculture and Natural Resources, Lilongwe, Central Region Malawi

**Keywords:** Community disaster resilience, Disaster risk management, Health, Malawi, Policy

## Abstract

Floods are among the most frequently occurring natural hazards in Malawi, often with public health implications. This mixed methods study assessed the capacity for and implementation status of the disaster risk management (DRM) strategy for the health sector in Malawi, using flooding in the Nsanje District as a case. Data were collected using desk review and a workshop methodology involving key officials from government ministries, national and international development partners, and the academia. The results show that Malawi had recently strengthened its DRM institutional frameworks, with a pronounced policy shift from reactive to proactive management of disasters. Health sector personnel and structures were key contributors in the design and implementation of DRM activities at all levels. Development partners played a significant role in strengthening DRM coordination and implementation capacity. Lack of funding and the limited availability, and often fragmented nature, of vulnerability and risk assessment data were identified as key challenges. Limited human resource capacity and inadequate planning processes at district level impeded full implementation of DRM policies. These findings call for community-level interventions for improved coordination, planning, and human resource capacity to strengthen community disaster resilience and improve public health. The approach used in this study can serve as a model framework for other districts in Malawi, as well as in other low- and middle-income countries in the context of Sendai Framework implementation.

## Introduction

Global disaster statistics for 2001−2018 revealed staggering economic damages at about USD 2 trillion and over 300,000 fatalities because of water-related disasters (Lee et al. 2020). Evidence suggests that these disasters will continue to increase in both magnitude and frequency (Phillips et al. 2015). Flooding is estimated to account for 40% of all natural hazard-related disasters worldwide causing about half of all deaths (Noji 1991; Ohl and Tapsel 2000). Notable and relatively recent water-related disasters include the tsunami in Southeast Asia in December 2004 (Ahern et al. 2005), cyclone Harold that hit Pacific countries during the COVID-19 emergency in April 2020, and Japan’s Typhoon Hagibis in 2019 (Ishiwatari et al. 2020). The African region is struck annually by natural hazard-related and human-made disasters, with direct and indirect impact on mortality, the disease burden, and health care delivery. For example, the 2010/2011 floods in Southern Africa affected about 150,000 people across nine countries and destroyed farmlands, housing, and social infrastructure including health facilities (WHO 2012).

The ability of communities to adapt to change, handle disruption, and respond positively and timely to emergencies in a manner that reduces impairment to its social, economic, health, and security functions, conceptualized as community disaster resilience (Cutter et al. 2008), is undermined by disasters such as floods. Nirupama (2013) argues that the effects of disasters could be significantly reduced if countries and communities identified, processed, and analyzed threats due to hazards, understood people’s vulnerability, assessed resilience and coping capacities of communities, and developed proactive strategies for future risk reduction—a process called disaster risk management (DRM). Aitsi-Selmi et al. (2015) further argues for the mainstreaming of health in DRM efforts as a way of addressing health inequalities and vulnerabilities that expose, mostly the poor, to the adverse effects of disasters such as flooding. A growing body of literature has, in many instances, established the nexus of health and disasters (Lechat 1979; Korteweg et al. 2010). These include disasters’ clinical and public health impacts (Lechat 1979; Korteweg et al. 2010), disaster epidemiology application (Malilay et al. 2014), emergency management and public health interactions (Clements and Casani 2016), and the role of public health in mitigating disaster risks (Shoaf and Rottman 2000). Despite this evidence, the centrality of health to mainstream disaster risk reduction (DRR) policies and practices has often not been recognized. Efforts to integrate health into DRR programs are reportedly scarce (Murray 2014), with the health sector maintaining a narrower clinical focus (Waring and Brown 2005).

Recognizing this gap, the World Health Organization (WHO) developed a DRM strategy for the health sector (WHO 2012), which in its preamble, recognizes health as the heart and missing link for effective DRM in the African region. The adoption of this strategy by WHO African Member States catalyzed the recognition of the centrality of the health sector in the management of disasters. Three years after the adoption of the WHO strategy, the Sendai Framework for Disaster Risk Reduction 2015−2030 (the Sendai Framework) further strengthened the need for integrating health in disaster risk responses. The strong emphasis on health in the Sendai Framework is demonstrated by its more than 30 explicit references to “health” in the document whereas its predecessor, the Hyogo Framework of Action 2005−2015 (HFA) mentioned “health” only 3 times (Maini et al. 2017). This focus on health was to ensure improved population health by linking individuals, systems, and communities with each other throughout the stages of a disaster, a concept called community health resilience (Wulff et al. 2015; Maini et al. 2017).

Almost a decade after the adoption of the WHO DRM strategy for the health sector, there are currently no publicly available assessments of DRM country capacity and implementation status against the nine targets set by WHO (Table 1). Using a consultative workshop methodology (Ørngreen and Levinsen 2017; Ahmed and Asraf 2018), participants from the government of Malawi (GOM), international and local development partners, and academics in Malawi were brought together at both the national and district levels to assess Malawi’s capacity and the status of implementation of the WHO DRM strategy for health. This study’s findings may inform not only future assessments in other districts of Malawi but could also serve as a model for low- and middle-income countries, particularly those in the African region, seeking to conduct similar exercises in the context of Sendai Framework implementation.

**Table 1:** World Health Organization (WHO) disaster risk management (DRM) strategy for the health sector targets and how they are linked to the domains of the WHO Country Capacity Assessment tool adapted for data collection for this study

WHO DRM Strategy Targets		Linkage with the WHO Country Capacity Assessment Domains
By the end of 2014 all Member States in the African region would have:		
(1)	Identified, assigned responsibility to, and equipped a unit in the MOH to coordinate the implementation of DRM interventions for the health sector;	Ministry of Health (MOH) coordination
(2)	Established functional health sector subcommittees in national multisectoral coordination committees on DRM;	Health sector coordination mechanisms
(3)	Incorporated DRM into their national health legislation, national health policies, and health sector strategic plans;	Institutional framework (policies, strategies, and legal frameworks)
(4)	Conducted health disaster risk analysis and mapping in a multisectoral approach.	Health emergency risk assessment and information management
By the end of 2017, at least 90% of Member States in the African region would have:		
(1)	Instituted a preparedness planning and management process that includes plan development, pre-positioning of essential supplies, resource allocation, simulations, evaluations, and annual updating based on all risks prevalent in the country;	Response and recovery operations readiness
(2)	Incorporated emergency and disaster early warning, preparedness, response, and recovery indicators into the national surveillance and health information systems;	Surveillance and information management
(3)	Instituted health facility and community resilience building, and preventive interventions based on disaster risk analysis and mapping;	Community support interventions
		Information, education, and communication
		Human resources
(4)	Established emergency and disaster response and recovery operations, based on national standard operating procedures, and capable of supporting cross-border interventions.	Response and recovery planning
By the end of 2022 all Member States in the African region will be fully implementing all the interventions of the Regional Strategy.		N/A

*Source* WHO (2012)

### Context of the Study

Floods and droughts are the most frequently occurring natural hazards in Malawi, accounting for an annual GDP reduction of about 1.7% (GOM 2019a). Malawi’s long history of weather-related disasters is also associated with poor health services and outcomes. For example, following the 2015 floods that affected 1,150,000 people, displaced 336,000, and killed 104 (GOM 2015a), the country experienced a surge in cases of malaria (23.1%), eye infection (8%), skin infection (39.9%), acute respiratory infection (19.9%), and diarrhea (18.2%), compared to a baseline year of 2013−2014 (GOM 2016). In addition, the floods damaged health facilities, available medical supplies failed to meet increased demand, and affected areas recorded high health worker absenteeism as staff homes were affected (World Bank 2015; GOM 2019b). In Nsanje District, which has an HIV prevalence of about 16% among the adult population, people lost their health passports in the 2015 floods, facilities experienced HIV drug stockouts, and many patients were out of treatment for up to two weeks (UNDRR 2015). The WHO DRM strategy for health seeks to improve the healthcare sector’s management of disaster risks, including the implementation of resilience building in health facilities and at community level (WHO 2012), an approach that has become even more relevant in the face of the recent COVID-19 pandemic with its attendant impact on health systems (Dzinamarira et al. 2020). The WHO DRM strategy for health sets nine targets (Table 1) for Member States to achieve by 2022 towards its full implementation.

Malawi is also a signatory to the International Health Regulations (IHR) 2005, which is a legally binding instrument requiring countries to develop, strengthen, and maintain the capacities to detect, assess, notify, and report public health events. Following the adoption of the DRM strategy for the health sector by WHO African Member States, Malawi made great strides in institutionalizing DRM as evidenced by the focus on reducing the socioeconomic impact of disasters in the Malawi Growth and Development Strategy (2012−2016) (MGDS) and the subsequent development of the country’s DRM policy in 2015. In general, DRM focus had reportedly shifted from response and recovery to DRR (GFDRR 2014). Similarly, and as evidenced by the Malawi National Resilience Strategy 2018−2030, Malawi’s DRM policy shifted from response and recovery towards the current focus on community resilience and early warning (GOM 2019c). However, only limited information is available on the actual shift in practice or the extent to which the policy has been implemented in Malawi, particularly at district and lower levels where adaptation to disasters occurs. Hence, this study sought to address the limited availability of information on the structural and institutional readiness for DRR in Malawi.

### Study Areas

**Fig. 1:**
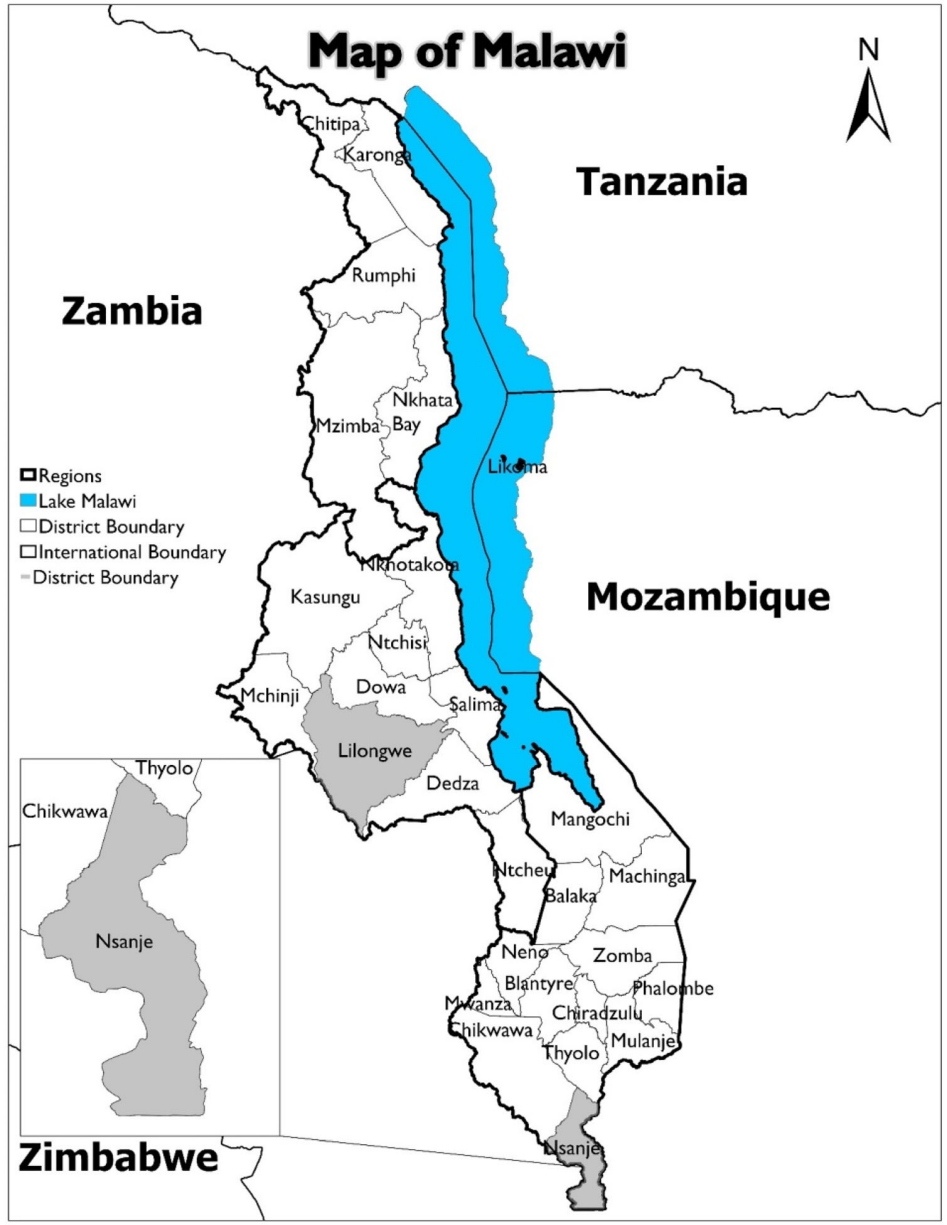
Map of Malawi showing the location of Nsanje District

This study was carried out in Malawi with national level assessments conducted in the country’s capital city, Lilongwe, and district level assessments conducted in Nsanje, the southernmost district of Malawi (Fig. 1). Nsanje lies in the Lower Shire River Valley, with the Shire River in the north and the rest of the district bordering Mozambique. The district covers an area of 1,942 km^2^ and has a population of 299,168 inhabitants. Nsanje has an estimated average terrain elevation of 241 m above sea level, with some hills in the southern-western part of the district rising to 610 m above sea level (GOM 2017a).

## Data and Methods

A mixed method facilitated consultative workshop approach (Ørngreen and Levinsen 2017; Ahmed and Asraf 2018) was adopted to collect both quantitative and qualitative data. The workshop methodology was deemed suitable for this study as, unlike other qualitative methods, it seeks to establish a shared position among participants after inter-subjective interactions (Ørngreen and Levinsen 2017). It enables collective problem-solving (Osborn 1948) through a participatory appraisal approach (Temu and Due 2000) comprising of group brainstorming and consensus building (Osborn 1953). A collectively agreed answer to each question is supported by evidence such as administrative and policy documents and examples of established institutions such as health subcommittees. The researchers were independent external reviewers/facilitators guiding the discussions towards mutual interactions and agreement on a common position, while ensuring there were no dominant voices. Collective positions were those considered to be accurate representations of the country’s capacity for mainstreaming health in DRM interventions and its implementation status of the various domains in the DRM strategy for health. This consensus building approach (Aitsi-Selmi et al. 2015) establishes a strong foundation for collective identification of gaps, and thus, agreement on required intervention pathways for effective integration of public health in DRM.

Cross-sectional quantitative data were also collected at the national and district levels (Nsanje District) during September and October 2019, respectively. Each of the WHO regional strategy target was mapped (Table 1) to the relevant domains of an adapted Country Capacity Assessment (CCA) questionnaire developed and implemented by WHO. The CCA adapted tool was administered to participants at both the national and district levels. In both instances, a workshop method was used for data collection, where participants gathered in one place to collectively review the questions and agree on the most appropriate response (an agreed group answer for each question) representing the country’s DRM capacity and implementation status, at the national and district levels, as at the time (Ørngreen and Levinsen 2017). At the district level, the data collection tool was adapted to ensure applicability by focusing on operational aspects of the strategy as opposed to high level policy issues. Consequently, domains one (institutional framework) and two (Ministry of Health coordination) were not assessed at the district level as they focused more on higher level policy and legislative aspects that were adequately responded to at the national level. Consensus scores at the national and district levels were averaged to result in scores reported in this study as described in the analysis section below. The questions in the CCA adapted tool required participants to collectively assess the availability, functionality, and operational status of institutional frameworks for DRM, health sector coordination, health disaster risk analysis and mapping, emergency and disaster early warning, disaster response and recovery, preparedness planning and management, and health facility and community resilience building.

At the national level, participants (*n* = 14) included staff from the technical subcommittees of the Department of Disaster Management Affairs (DODMA) (*n* = 5), the Ministry of Health (MOH) (*n* = 1), the World Bank (WB) (*n* = 1), the World Food Programme (WFP) (*n* = 1), the Ministry of Water Affairs (*n* = 1), the Department of Climate Change and Meteorological Services (DCCMS) (*n* = 1), the Housing Department (*n* = 1), the Environmental Affairs Department (EAD) (*n* = 1) and the Centre of the Lilongwe University of Agriculture and Natural Resources (LUANAR) (*n* = 2). At the district level, participants (*n* = 20) included staff from the District Executive Council (DEC) representing the various committees responsible for DRM implementation (*n* = 6) and nongovernmental organization (NGO) representatives (*n* = 14).

### Quantitative Data Analysis

The questions were assessed on a scale of ordered response options. Most of the questionnaire items (181 out of a total of 225) had the response options “Yes completely” (coded 2), “Partially” (coded 1), “No, not at all” (coded 0), and “Don’t know” (to be excluded from analysis). One question inquired whether health sector DRM related training had been conducted, with response options “Yes” (coded 1) and “No” (coded 0). There were 4 questions requiring participants to indicate when tabletop exercises and disaster management simulations were conducted and had response options “In the past year” (coded 3), “In the past 2 years” (coded 2), “In the past 3 years” (coded 1), and “Don’t know” (to be excluded). A question inquiring on the development status of the health sector plans addressing DRM had five questionnaire items, scored as follows: “Completed and coordinated with national disaster office” (coded 3), “Completed” (coded 2), “Being developed” (coded 1), “To be developed” (coded 0), and “Don’t know” (to be excluded). A similar question, with 13 questionnaire items, inquired on the development status of health sector related DRM policies, with the following response options: “Completed and approved” (coded 3), “Completed but not approved” (coded 2), “Being developed” (coded 1), “To be developed” (coded 0), and “Don’t know” (to be excluded). Four questionnaire items required participants to rate different health sector DRM structures/committees and hazard information according to their perceived level of functionality and accessibility, respectively. These items had response options “Very low” (coded 0), “Low” (coded 1), “Adequate” (coded 2), “High” (coded 3), and “Very high” (coded 4). There was one questionnaire item that required participants to indicate their level of agreement with the statement that the health disaster coordinator had enough resources to lead the health sector DRM program. This question’s response options were coded as follows: “Strongly agree” (coded 2), “Agree” (1), “Disagree” (0), “Strongly disagree” (0), and “Don’t know” (to be excluded). None of the questions returned a “Don’t know” collective response. Hence, no questionnaire item was excluded in all instances that this was a response option.

**Fig. 2:**
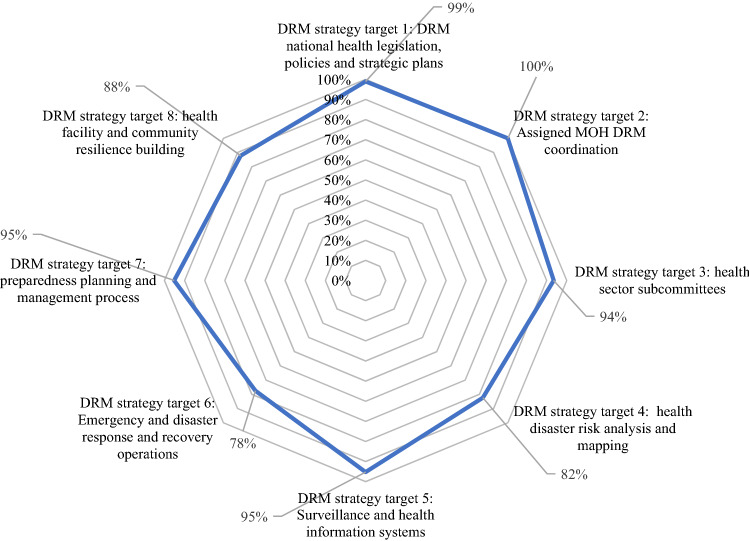
Average percentage scores achieved for each of the African Regional Strategy for Health targets as at October 2019

Quantitative data analysis was conducted using a Microsoft Excel database (Olu et al. 2016). Each questionnaire item was assigned a respective numeric score (as described above) to calculate the mean scores for each domain and its sub-domains that match the respective DRM health strategy target. The following analysis steps were conducted:

- Each WHO regional strategy target was mapped to the adapted CCA questionnaire domain (see Table 1).^1^
- After obtaining national and district level scores separately, composite consensus scores for each questionnaire items were determined by gathering evidence that supported each score. Such evidence included available policy documents, minutes of meetings, training attendance registers, among others.
- It was possible to obtain different scores at the national and district levels regarding the conduct of activities such as simulations and tabletop exercises if these were not conducted by national stakeholders but by NGOs operating at the district level. In such instances, both scores were averaged and recorded as such.
- After obtaining a composite consensus score, categorical Likert scale responses for questionnaire items were converted into respective numeric scores (through coding) for each response to the survey questions as described above.
- Individual scores for each questionnaire item making up a sub-domain were summed to obtain the score for the respective sub-domain. This score was used as the numerator in percentage calculation of the extent of sub-domain implementation, with the maximum obtainable score for that sub-domain as the denominator.
- Where one sub-domain matched a domain, the sub-domain total score was used as a numerator (N) in the percentage calculation of the extent of domain implementation.
- Where there was more than one sub-domain making up a domain, an average of the sub-domains was calculated to obtain the score for the respective domain that was subsequently used as a numerator (N) in the percentage calculation of the extent of domain implementation.
- The maximum possible score for each questionnaire item, depending on the response options and coding, ranged between 1 (for example, on a “Yes” and “No” Likert scale) and 4 (on a “Very low,” “Low,” “Adequate,” “High,” and “Very high” Likert scale). The sub-domain maximum possible score used as a denominator (D) in the percentage calculation of the extent of sub-domain implementation was calculated by multiplying the number of questionnaire items in each sub-domain by the maximum possible score for the respective question response type that make up the sub-domain.
- The percentage score for each domain was obtained by dividing the numerator by the denominator (N/D) multiplied by 100 (Fig. 2).

A score of ≥ 90% was considered adequately achieved/implemented as the regional strategy targets were supposed to have been completely achieved by 2017.

### Qualitative Data Analysis

Qualitative data were collected in two ways: First, the consultative workshops conducted with key informants representing various stakeholders discussed above generated qualitative explanatory information on the collective answer to each question. Supporting qualitative quotes are provided to substantiate common positions as agreed by participants in addition to supporting administrative and policy documents and referenced institutional structures such as existing technical subcommittees. Second, available operational policy documents, declarations, resolutions, guidelines, and reports associated with DRM implementation after 2012, when the regional strategy was adopted, were assessed. Information obtained from document review was used to substantiate and validate findings from the consultative workshops. Table 2 presents the approach used for document review.

**Table 2:** Approach for reviewing Malawi’s disaster risk management (DRM) related literature

Documents accessed and reviewed	Aspects searched for in the documents
Disaster Risk Management: A Strategy for the Health Sector in the African Region (2012) Capacity Development Plan 2017/2018–2019/2020 for the Malawi Department of Disaster Management Affairs Malawi National Disaster Risk Management Policy 2015 (GOM 2015b) National progress reports on the implementation of the Hyogo Framework for Action (post 2011–2013 and 2013–2015) Sectoral policies and strategic plans (by technical subcommittee) Post Disaster Needs Assessment reports (2012–2019) Malawi National Resilience Strategy (2018–2030) Assessment Report on Mainstreaming and Implementing Disaster Risk Reduction Measures in Malawi (2015) Disaster Risk Financing Strategy and Implementation Plan (2019–2024) Nsanje District Council initial assessment report of March 2019 flood situation (2019) Published research papers National policies and plans (currently under implementation) Official statements and presentations by government officials Reports from development stakeholders Documents of meetings and DRM conferences in Malawi Documents of DRM trainings in Malawi	*Explicit mention of key DRM issues* Community resilience in the context of risk management Managing the risk of flooding and its effects Role of the Ministry of Health and other disaster coordination structures Community participation in policy formulation and implementation Access to health and social services before, during, and after disasters Disaster risk reduction systems and structures Role of district and community level structures Role of development partner (NGO) stakeholders Operational status of each of the identified policies, systems, and structures post formulation For the above, the year of the policy statement, event occurrence, and document publication was identified to ensure that it aligns with the development and adoption of the African Regional Strategy for Health

*Source* Adapted from Bowen (2009)

Thematic analysis (Casteleberry and Nolen 2018) was used to analyze qualitative data obtained from the consultative workshops. A deductive analysis approach was used in which the nine targets of the regional strategy and its components were used as preconceived themes and sub-themes. Data were categorized under each of these themes and sub-themes and analyzed to provide an explanatory understanding of the quantitative scores assigned by the participants based on their perceptions of capacity for and implementation status of the African Regional Strategy for Health.

Findings from the document review were analyzed using the policy triangle framework developed by Walt et al. (2008) and further enhanced specifically for the health sector (O’Brien et al. 2020). Using this framework, the analytical procedure of this study focused on the context (mostly disaster occurrence) informing the need for the policy; the explicit concern about DRM in the policy, for example, mention of shifts from a reactive to a proactive DRM approach and financial commitments (content); the participants in the formulation and implementation process to investigate inclusion of stakeholders, including those from districts and the international community (actors); and the adopted policy implementation process, focusing on the rollout plan and resource commitment (process).

## Results

This section presents the results from data analyses conducted to assess capacity and implementation status of the DRM strategy for health and community disaster resilience. The section is presented using the DRM strategy for health targets as themes. Using this approach, the section presents the high-level institutional framework arrangements such as the role of the Ministry of Health followed by specific subnational level operational issues such as health facility and community resilience building intervention.

### Disaster Risk Management Institutional Arrangements in Malawi

In terms of policy context, the review of documents revealed that there is a DRM structure in place, called the Department of Disaster Management Affairs (DODMA), established by the Disaster Preparedness and Relief Act of 1991 (GOM 2010). Its purpose is to coordinate all DRM activities in the country. The DODMA is made up of two divisions: (1) the disaster risk reduction division, focusing on coordinating the implementation of DRR programs, and (2) the disaster response and recovery division, which is responsible for coordinating the implementation of disaster response and recovery programs.

Existing policies and strategic documents pertaining to DRM and in support of the African Regional Strategy for Health include the National Social Support Policy (2012), the National Climate Change Investment Plan (2013−2018) (2013), the National Adaptation Program for Action (Revised, 2015), the National Disaster Recovery Framework (2015), the National Climate Change Management Policy (2016), the Malawi Growth and Development Strategy III (2017−2022) (2017), and the Agriculture Risk Management Strategy (2017−2022) (2017). Development of these policies followed the adoption of the Hyogo Framework of Action 2005−2015 (HFA) that emphasized DRR. At the time of data collection, it was reported that a draft Bill revising the 1991 Act had been approved by the Cabinet and was awaiting parliamentary approval. Provisions within all these policies draw clear links between the health outcomes of disasters and the implementation of DRM activities.

The capacity assessment investigated the operational environment of the DODMA that has a direct bearing on DRM capacity and implementation status in Malawi. Table 3 presents quantitative sub-domain scores for the items assessed for each of the WHO African Regional Strategy for Health targets.

**Table 3:** Summary of averaged achievements against disaster risk management (DRM) for African Regional Strategy for Health targets as at October 2019

DRM for health strategy target	Questionnaire domain	Questionnaire sub-domain	Questionnaire sub-domain scores and % achieved
DRM strategy target 1: Incorporated DRM into their national health legislation, national health policies, and health sector strategic plans	Institutional Framework (Policies, Strategies, and Legal Frameworks)	Legal framework	16/16 (100%)
		Policy framework	48/49 (98%)
DRM strategy target 2: Identified, assigned responsibility to, and equipped a unit in the MOH to coordinate the implementation of DRM interventions for the health sector	Ministry of Health (MOH) Coordination	MOH DRM coordination role	8/8 (100%)
DRM strategy target 3: Established functional health sector subcommittees in district multi-sectoral coordination committees on DRM	Health Sector Coordination Mechanism	Health sector subcommittees’ functionality	72/77 (94%)
DRM strategy target 4: Conducted health disaster risk analysis and mapping in a multi-sectoral approach	Health Emergency Risk Assessment and Information Management	Hazard assessment	3/4 (75%)
		Vulnerability assessment	12/12 (100%)
		Risk assessment	13/18 (72%)
DRM strategy target 5: Incorporated emergency and disaster early warning, preparedness, response, and recovery indicators into the district surveillance and health information systems	Surveillance and Information Management	Health information system	12/14 (86%)
		Surveillance system	10/10 (100%)
		Rapid health needs assessment	6/6 (100%)
DRM strategy target 6: Established emergency and disaster response and recovery operations, based on national standard operating procedures, and capable of supporting cross-border interventions	Response and Recovery Planning	Planning framework	8/10 (80%)
		Planning process and plan content	45/60 (75%)
DRM strategy target 7: Instituted a preparedness planning and management process that includes plan development, pre-positioning of essential supplies, resource allocation, simulations, evaluations, and annual updating based on all risks prevalent in the country	Response and Recovery Operations Readiness	Health system institution/facility level readiness	3/3 (100%)
		Logistics and surge support readiness	47/52 (90%)
DRM strategy target 8: Instituted health facility and community resilience building, and preventive interventions based on disaster risk analysis and mapping	Community Support Interventions	Community level risk assessment	7/10 (70%)
		Community level preparedness	9/10 (90%)
		Community level DRM structure	10/10 (100%)
	Information, Education, Communication	Communication strategies	18/18 (100%)
		Pre-/Post-event DRM related public health awareness	30/32 (94%)
	Human Resources	Human resource capacity development	19/26 (73%)
DRM strategy target 9: By the end of 2022 all Member States in the African region will be fully implementing all the interventions of the African Regional Strategy for Health^a^	All	All	N/A

Sub-domain scores obtained are displayed as numerators and the maximum obtainable from adding up scores from items making up a particular sub-domain are displayed as denominators

^a^Target not assessed as it was only due in 2022

Overall, the participants scored the country and district performances highly across most of the assessed sub-domains, with about 40% of the sub-domains scoring 100%. The data in Table 3 show that hazard assessment (75%), risk assessment (72%), planning process and plan content (75%), health information system (86%), community level risk assessment (70%), and human resource capacity development (73%) were the least performing (< 90%) of all the variables assessed. Figure 2 presents the percentage scores achieved for each of the WHO African Regional Strategy for Health targets as at October 2019.

Figure 2 shows that the country did well in terms of meeting targets that relate to establishing policies and coordination mechanisms, with scores of about 90% and above. Scores of below 90% were recorded for targets relating to DRM operationalization, most of which is at the district level (Table 3).

### Ministry of Health Disaster Risk Management Coordination Role

The study found that the Malawi Disaster Preparedness and Relief Act of 1991, which was the guiding Act for all DRM work in Malawi at the time of the investigation, refers to “health” twice. First, it provides for the inclusion of the Secretary of Health in the National Disaster Preparedness and Relief Committee of Malawi as an ex-officio member. Second, it directs the Minister responsible for all DRM work to consult with the Minister of Health regarding burials during disasters.

As part of the DODMA structure and DRM policy coordination actors, the MOH is mandated with leading the health and nutrition technical subcommittee. This structure is replicated at all lower-level government tiers with the District Health Officer (DHO) and the District Environmental Health Officer (DEHO) being part of the District Health Team (DHT) and the District Executive Council (DEC) responsible for all DRM work at the district level. It is through this technical subcommittee that health sector specific DRM work is implemented. The following quote is illustrative: “The policy mandates every cluster, so nutrition is part of it, health is also part of it and agriculture” (Participant, National Consultative Workshop).

The main challenge reported in relation to the capacity of the MOH, and all other clusters, is the unavailability of funding to conduct DRM activities on a continuous basis. The following quote is illustrative: “The budget is there but it is empty, it doesn’t have money and it goes year in, year out” (Participant, National Consultative Workshop).

### Establishment of Functional Health Sector Subcommittees

In terms of the national DRM institutional structure, this study found that a National Health Disaster Coordinator is appointed reporting to the cluster lead in the national DRM structure and the Director of the MOH. A health and nutrition technical subcommittee was also established responsible for providing health DRM advisory functions to the national disaster management committee. The chair of the health and nutrition technical subcommittee falls under the MOH, thereby enabling the mainstreaming of DRM functions in the programs of the parent ministry. At the district level, the subcommittees are represented by the DHOs and the DEHOs.

### Disaster Risk Management Incorporation into National Health Legislation, Policies, and Plans

This study found that the health sector in Malawi is guided by the Public Health Act of 1948, as amended, the National Community Health Strategy (2017−2022) and the National Health Policy of 2017. In terms of policy content, only the National Health Policy explicitly mentions DRM. Outlined within the National Health Policy (2017) is a priority area on social determinants of health, which includes, as one of its strategies, the need to strengthen disaster, outbreak, and epidemic preparedness and response. By including disaster preparedness, this strategy is in line with the new DRM policy (2015b).

The document review found that, at the time of data collection, the Disaster Preparedness and Relief Act (1991) was the guiding Act and it provided the legal framework for all DRM work in Malawi. The study also found that the country had a Disaster Risk Management Bill no. 13 of 2019 that had recently been approved by the Cabinet, but had not yet been fully endorsed by the Parliament (World Bank 2018). According to the national workshop participants, this new Bill represented a shift from crisis management to a more proactive and comprehensive risk management approach.

The study found that, in 2015, the GOM developed the National Disaster Risk Management Policy (2015b) with support from NGO partners. The policy defines how the country will coordinate the implementation of DRM activities. Operational guidelines were reported to have been developed to operationalize the DRM policy. The guidelines outline the responsibilities of different role players from the district to national levels.

### Health Disaster Risk Analysis and Mapping

This target focuses on health emergency risk assessment and information management, with emphasis on risk identification, vulnerability assessment, and risk assessment. District level participants reported that useful information on flooding was clearly defined, readily available from the national repository, and provided to planners in understandable formats, albeit mostly in hard copy. The following quote is illustrative: “That information is available, but it’s a paper-based information” (Participant, District Consultative Workshop).

The document review, however, revealed that the last country-wide risk assessment and mapping for drought and floods, the Economic Vulnerability and Disaster Risk Assessment Review in Malawi, was conducted by the DODMA in 2009, with support from the World Bank (GFDRR 2009). While a district health risk assessment was conducted in Nsanje, the participants, mainly from the NGOs, reported having conducted supplementary local and fragmented community level vulnerability assessments to inform their program needs. The following quote illustrates this point: “Some areas are yet to come up with vulnerability assessments pertaining to various hazards. If I want to implement an intervention in TA Nyachikadza as an NGO I can just go there and make a partial vulnerability assessment” (Participant, District Consultative Workshop).

### Preparedness Planning and Management Process

The study found that Nsanje District officials had, together with district level multi-sectoral stakeholders, developed a plan to operationalize the national DRM policy. In terms of process, the district council, working with NGOs, coordinates the purchase of supplies for disaster preparedness and planning. Systems and mechanisms for managing and distributing medical supplies are in place through the decentralized health facilities. All health communication is done through the District Health Promotion office that utilizes various platforms, including the civil protection committees and their representatives at the village and area levels. The participants reported that the district council had no surge capacity for ambulance services in times of disasters but relied on NGO partners to provide more ambulances when needed.

In terms of logistical resources and support needed for flooding, medical supplies and equipment to pre-hospital activities, hospital, temporary health facilities, and public health were reportedly well coordinated, readily available, and periodically tested according to established guidelines. Procedures for procurement of exceptional supplies were reported to be in place and the cold chain for medical supplies was maintained. Pharmaceutical services were also reported to be in place and readily available. The availability of these services was, however, based on available and yet fragmented place-specific risk analysis conducted by individual NGOs at the community level. The study did not find procedures for the pre-positioning and release of essential supplies to high-risk areas. The participants also reported that there was limited capacity for maintaining life support while transporting patients from disaster affected areas as well as for management of medical activities on the disaster scene.

### Emergency and Disaster Early Warning, Preparedness, Response, and Recovery Indicators

As most non-state actors rely on initial rapid assessment (IRA) reports for fund raising and activity implementation, they support the conduct of IRAs during and immediately after a disaster. The participants reported that the District Health Information System (DHIS) was used for health data management with thresholds/triggers for switching from routine to emergency mode. The district rapid response team was reported to be well trained. However, the participants reported that the surveillance function in the DHIS was managed manually at the facility and community level. In addition to the electronic DHIS surveillance system, the participants reported that unconventional early warning systems (EWS) exist at the village level. Examples given included river gauges, rainfall forecasts, and the use of indigenous knowledge. The following quote is illustrative: “We also have indigenous knowledge e.g. when we see ants coming out in large numbers in November, it’s an indication that there will be a lot of rainfall. When we see a lot of mangoes in the trees, we anticipate drought” (Participant, District Consultative Workshop).

### Health Facility and Community Resilience Building, and Preventive Interventions

This target relates to the availability and functionality of community support interventions, information, education and communication, and human resource capacity development. This study found that DRM structures existed and were functional at the community level. Below the district in each Traditional Authority (TA) were Area Civil Protection Committees (ACPCs), and below those, were Village Civil Protection Committees (VCPCs). In addition, it was reported that there are Area Development Committees (ADCs) responsible for coordinating all development work in an area. There were clear terms of reference (TORs) developed by the DODMA for these structures.

In terms of risk and vulnerability assessment, it was reported that communities are fully involved in the process, and they understand the parameters of risk and vulnerability as they participate in the data collection. Community participation was, however, mostly pronounced in local areas of interest to implementing partner NGOs. Community level preparedness is coordinated through the VCPC volunteers who work closely with local clinics and the police. Resources for response are kept at local health facilities and police stations.

Community radio, megaphones, whistles, drums, and cellphones were used as media for communicating early warning messages. Pre-established communication mechanisms were reported to be in place and defined in the district plan. These outline how to care for vulnerable groups like the elderly, sick, and children in the case of flooding. Pre-flooding public awareness raising was conducted using Information Education and Communication (IEC) materials that are available in local languages. The level of public awareness was, however, not systematically and regularly measured to inform the development of awareness information.

In terms of human resource capacity development, a capacity development plan was reported to be available among NGO partners. Training offered was based on available skilled human resources and not on competencies required for a specific hazard. The participants reported that a database of all trained staff exists in hard copy. The process of accessing training funds from the DODMA was reported to be cumbersome and discouraging.

### Emergency and Disaster Response and Recovery Operations

This study found that the various subcommittees of the DEC, including health, developed individual plans that were then consolidated into one plan, called the district contingency plan. After the district plan is developed, it is submitted to the DODMA for review and approval before implementation. The plan covers aspects such as coordination of international humanitarian assistance based on national standards, health management in shelters and temporary settlements, identification and handling of dead bodies, objectives and actions of recovery, considerations of vulnerable groups and logistical arrangements, among others. To ensure business continuity in cases of flooding, mobile clinics are deployed into communities to provide services. At the community level, it was reported that the availability of disaster plans varied from one place to the other: “We have just conducted a baseline, most of the disaster risk management plans at community level are mostly done by partners when they want to conduct a project, they would at least gather a community and develop the village action plan. So, it varies from one area to another” (Participant, National Consultative Workshop).

There were no reported simulations and tabletop exercises conducted at the district council level, except at the community level where NGOs operated. The following quote is illustrative: “Yes, at community level we have done the drills and simulations. But now at district level, we have never had any simulations” (Participant, District Consultative Workshop).

## Discussion

This section is divided into three subsections focusing on DRM institutional structure in Malawi, its financing, and how it is operationalized at the district and community levels.

### Disaster Risk Management Institutional Structure

This study has shown that Malawi has made significant strides towards strengthening its DRM capacity and meeting the African Regional Strategy for Health targets. Despite having well developed institutional structures, including a DRR division within the DODMA, the government approach to DRM remained reactive due to limited investment in preparedness activities.

As noted by the World Bank (2019), the lack of pre-financing for preparedness and a reactive approach to DRM undermine the DRR functions of the DODMA. This lack of preparedness has resulted in disasters with increasing and significant impact on people’s lives (GOM 2019a), and regular post-disaster emergency appeals, which have received relatively little budgetary contribution from the government (Mijoni and Izadkhah 2009). Government funding for preparedness remained low (Manda 2014) and disasters were seemingly treated and accepted as part of life with dire consequences for populations at risk. As noted by Clary (1985) and Ng’oma and Mwamlima (2008), crises continued to invoke government action and informed policy formulation, with action only coming after the occurrence of a disaster event.

Analysis of the policy development context, process, and environment shows that Malawi benefited from international policy instruments such as the HFA. The HFA reports (2013−2015) and GOM official statements show that the country started shifting from a reactive disaster management approach to an all-inclusive DRM approach in part due to the requirement for reporting on the achievement of HFA targets to the United Nations (UN). The HFA sought to ensure that DRR is a national strategy for reducing disaster underlying risk factors. Therefore, to align with this international framework for DRR and the WHO African Regional Strategy for Health, the GOM developed policies and strategies upholding risk management as a gold standard. Examples of such instruments include the Health Policy (2017), the National Community Health Strategy (2017−2022), the National Resilience Strategy (2018−2030), and the National Disaster Risk Financing Strategy and Implementation Plan (2019−2024). In its acknowledgment of the DRM political commitment by the GOM, the World Bank (2019) highlighted the surge in the development of policies that mainstream community resilience strengthening between 2012 and 2019.

The establishment of health sector subcommittees through the decentralized DODMA structures provides the ministry space to lead all health sector DRM activities in the country. Despite this progress in building a strong foundational legislative, institutional, and policy framework, there are challenges that may hinder the country from achieving a fully implemented regional strategy by 2022 (target 9). From a legislative perspective, the delayed finalization of the revised Disaster Act, to replace the outdated 1992 Act, limits the full implementation of the 2015 DRM policy. In addition, inadequate financial capacity, and limited availability of comprehensive risk assessment data, both at the national and district levels, affect the country’s ability to effectively coordinate policy implementation.

### Financing the Disaster Risk Management Institutional Structure

The typologies of financial instruments for both ex ante and ex post DRM activities in Malawi reflect and confirm what Goldsmith and Eggers (2004) and Milward (1996) have called “hollow states”—states that rely on development partners for joint or singular delivery of public services. This study found that DRM ex ante funding is drawn from a budget vote on unforeseen expenditure, which does not exceed 2% of the total budget, and is disbursed not to exceed the available balance at the time of need (GOM 2017b). For ex post activities and to mitigate the adverse effects of disasters, the government relies on budget reallocations, post-disaster borrowing, external assistance, post-disaster support to the affected, and scalable social protection programs (GOM 2017b).

This financial structure and level of commitment reconfirms the historically reactive and crisis-driven approach of governments to DRM. This observation is not unique to Malawi. The UN Office for Disaster Risk Reduction’s (UNDRR) Global Assessment Report on Disaster Risk Reduction 2019 reports on a multi-country assessment conducted in Cameroon, Ghana, Malawi, and Senegal showing that developing countries lack financial resources and financial planning capacities for DRM (UNDRR 2019). The Malawi HFA report (2011−2013) also identified the lack of DRR funding from the central government to the DODMA as the major limiting factor in DRM implementation. For example, Kita (2017a) notes that in 2015/2016, the DODMA had a total budget of USD 125,000 against a total drought impact estimated at USD 365.9 million. The lack of adequate resources for DRM implementation is a major disincentive for the implementation of DRM activities at the community level as exemplified by the relatively lower scores (< 90%) achieved against targets that are more applicable at lower levels. Reliance on short-term donor funding does not allow for full operationalization of resilience focused institutional frameworks developed by the country. A shift from disaster response to DRM needs to be accompanied by a move from short-term donor funding to multi-year DRM financing, which supports the scaling up of activities that strengthen disaster risk resilience at the community level where adaptation to disasters occurs.

### Operationalizing Disaster Risk Management at the District Level

The study found that Nsanje DEC, as the nucleus of DRM implementation in the district, had gained considerable experience in coordination, control, and monitoring DRM activity implementation by various stakeholders. The strength of their control lay in two main factors, the first being the existence and functional state of the district-controlled DRM civil protection committees. The second is the district’s centralized control of all external humanitarian and emergency funding and supplies through its health facilities and the police service. The coordination ability was also demonstrated by the availability of a district DRM plan consolidating individual sector plans developed with the help of NGO partners. The integration of health activities is achieved through the participation and leadership of the DHO and DEHO in the DEC, as well as coordination of all communication through the district health promotion office. It was observed, however, that operationalization of DRM was generally based on inadequate risk assessments. Assessments that were done were carried out by NGO partners, often in a fragmented manner at the community level and in areas of interest to them.

Despite the strengthened institutional capacity for implementation of DRM interventions, this study revealed that lack of resources at the local government level often resulted in the incapacitation of these structures unless they were supported by NGO partners. This observation was supported by the GOM in its Disaster Risk Financing Strategy and Implementation Plan (2019), in which it highlighted that most of the local authorities receive about 2% of the national budget against a legislated 5% due to financial constraints at the central government level. As a result, most of the community-based DRM activities were implemented by NGOs who had time-bound objectives and donor focused reporting requirements. This often results in fragmented and suboptimal implementation of critical resilience building DRM activities at the community level. For example, trainings were reportedly not informed by competence needs. Available risk assessment datasets were mostly in hard copy format, which made access and sharing with stakeholders cumbersome. These findings raise questions on how the district manages to implement DRM activities.

It was apparent from the consultative workshops that NGOs had taken a lead in supporting the DEC in DRM design and implementation. This was mainly because NGOs had emergency funds to support the district at the onset of, during, and immediately after a disaster. While this support made the operationalization of district DRM activities possible, it presented its own challenges. First, most of the donor funding was short-lived and focused on response activities, which undermined the DEC’s ability to initiate and implement any resilience strengthening preparedness activities. This finding is consistent with the observation made by the GOM in the HFA report (2011−2013) that donors were not supporting preparedness activities. Second, donor funded projects supported data generation activities only in times and/or geographical areas of their interest, and not district-wide collection of vulnerability and risk assessment data. This meant that resilience strengthening activities were fragmented across the district and dependent on NGO priorities. Third, the reliance on NGO partners for DRM activity implementation created a donor dominance and dependence, establishing a structure that Kita (2017b, p. 246) called “Third-party government,” which refers to the delivery of public services by NGO partners. In addition, Trogrlic et al. (2018), concluded from their study in the Lower Shire Valley, that flood risk management strategies often fail because NGOs do not have exit strategies and thereby fail to translate ownership of interventions to communities. This observation was also supported by the findings of this study.

In line with findings by Tiepolo and Braccio (2020) that poor DRM plan preparation capacity limits the implementation of DRR actions, this study found consistent underperformance in the areas of planning process, plan content, and in relation to human resource capacity development at a community level. In addition, given that vulnerability and risk assessment underlie successful implementation of well-developed DRM plans (OECD 2012), the suboptimal performance observed in this study in these areas could potentially explain the consistently high disaster losses experienced by Nsanje District communities following a disaster.

## Study Limitations

This study was limited to two participatory workshops, one conducted at the national level and another in one district of Malawi. The participation of the DODMA in these workshops may have influenced how the other participating organizations’ representatives responded to or agreed with the scores provided. However, requests were made for supporting documents to substantiate suggested scores, thereby validating the scoring. In addition, the workshop conducted at the national level and the reflections shared on national DRM policies and practices helped to ensure generalizability of study findings, with a caution that there could be district performance differences.

## Conclusion

This study revealed that Malawi has made significant progress towards the establishment of a strong institutional framework for DRM implementation, particularly as it relates to development of policies and coordination mechanisms. This is despite the country not meeting all the 2014 and 2017 targets set out in the WHO African Regional Strategy for Health. Within its coordination arrangements, the country has placed the MOH as the lead for all health-related DRM activities, from the national to the district level. The inclusion of disaster preparedness and adaptation as one of the objectives in the current national health policy is evidence of government efforts to integrate DRM activities in the health sector. The main hindrances to strengthening capacity for DRM implementation are limited preparedness, suboptimal risk assessment, and inadequate funding allocation to DRM activities. As a result, DRM seems to be stronger on paper and intent, but weak in practice as observed at local levels where adaptation to disasters occurs.

This study concludes that to ensure effective and full implementation of the WHO African Regional Strategy for Health, the government, with support from non-state actors, should develop a cost-effective financial model that makes funding available for disaster preparedness and mitigation, including ensuring capacity for comprehensive risk and vulnerability assessments. As the world aims for full operationalization of the Sendai Framework, the results from this study suggest that the development of policies and establishment of institutions incorporating health need to be supported by similar community resilience strengthening interventions that are informed by data that identifies the vulnerabilities of disaster-prone communities. Strengthening community health systems should be at the center of such interventions as healthy communities are better able to adapt to disasters. Although this study was focused on flooding as the hazard, and Nsanje District as the study site, some of its findings are applicable to other hazards such as drought or disease outbreaks. The findings are also applicable to other districts as they are guided by the same pieces of legislation, which are implemented and coordinated by the same structures at the national level and through similar arrangements at the local level. Therefore, the approach used in this study for assessing the implementation status of DRM strategy for the health sector can serve as a model framework for other districts in Malawi, as well as for other low- and middle-income countries in respect of the implementation of the Sendai Framework.

## Footnotes

1 Number of questionnaire items assessed for each domain: institutional framework = 26; Ministry of Health coordination = 4; health sector coordination mechanism = 36; health emergency risk assessment and information management = 16; surveillance and information management = 15; response and recovery planning = 33; response and recovery operations readiness = 27; community support interventions = 14; information, education, and communication = 25; human resources capacity development = 29.
